# 非小细胞肺癌合并左房瘤栓病例的手术治疗与围术期管理

**DOI:** 10.3779/j.issn.1009-3419.2018.01.04

**Published:** 2018-01-20

**Authors:** 彤 鲍, 飞 肖, 德若 刘, 永庆 郭, 朝阳 梁

**Affiliations:** 100029 北京，中日友好医院胸外科 Department of Thoracic Surgery, China-Japan Friendship Hospital, Beijing 100029, China

**Keywords:** 肺肿瘤, 瘤栓, 体外循环, 左房切开取栓术, Lung neoplasms, Tumor thrombus, Cardiopulmonary bypass, Atriotomy

## Abstract

**背景与目的:**

非小细胞肺癌合并左房瘤栓病例在局部晚期肺癌中占有一定比例，积极外科手术能否带来获益，以及具体术式选择均存在争议，是目前外科研究的热点。我们报告了单中心接受手术治疗的非小细胞肺癌合并左房瘤栓病例队列，结合预后分析探究合理的诊疗方法。

**方法:**

自2006年8月-2017年7月共有11例非小细胞肺癌合并左房瘤栓病例在中日友好医院胸外科确诊并行手术治疗，对其临床资料、治疗选择、病理类型、预后情况进行了回顾性研究。

**结果:**

11例患者中，男性7例，女性4例，平均年龄57.9岁，6例患者接受术前新辅助放化疗。全部患者手术过程顺利，其中行正中开胸体外循环下手术3例，体外膜肺氧合辅助下后外侧切口入路手术1例，常规后外侧切口入路手术6例，胸腔镜辅助小切口入路手术1例。手术达R0切除9例，R1切除2例。手术用时210 min-380 min，平均292 min，出血量100 mL-1, 600 mL，平均436 mL。全组有1例（9.1%）术后90天内死亡病例，另有4例（36.4%）出现心律失常、脑梗、低氧血症等围术期并发症。术后病理诊断鳞癌6例，腺癌4例，肉瘤样癌1例，病理分期pT4N0M0 7例，pT4N1M0 4例。术后对9例患者行辅助化疗，随访期内对2例患者行放射治疗。全组随访时间2个月-53个月，3年无病生存率30.7%，中位无病生存期31个月，3年总体生存率49.1%，中位总体生存期为33个月。

**结论:**

对有选择的非小细胞肺癌合并左房瘤栓患者，选择合理术式切除肿瘤病灶和肺静脉、左房内瘤栓，加强围术期管理并配合新辅助/辅助化放疗，可能获得满意的预后。

## Abstract (en)

**Background and objective:**

Non-small cell lung cancer with left atrial tumor thrombus accounts for a small proportion of local advanced lung cancer. Whether surgery could bring benefits, as well as surgical options are still controversial, and have always been hot spots in surgical research. We report a single center experience of surgical treatment to non-small cell lung cancer with left atrial tumor thrombus, aim to figure out more reasonable treatment strategy.

**Methods:**

From August 2006 to July 2017, a total of 11 cases of non-small cell lung cancer with left atrial tumor thrombus underwent surgery in Thoracic Surgery Department of China-Japan Friendship Hospital. Clinical data, treatment options, pathological types and prognosis of these patients were collected to perform a retrospective study.

**Results:**

Of the 11 patients (mean age of 57.9), 7 were men and 4 were women. Six of them received neoadjuvant radiotherapy and/or chemotherapy. All patients underwent smooth operation, including 3 cases with cardiopulmonary bypass, 1 case of posterolateral approach under extracorporeal membrane oxygenation, 6 cases of conventional posterolateral approach and 1 case of video-assisted minithoracotomy. Nine patients were evaluated as R0 resection while 2 cases were evaluated as R1 resection. The Surgeries cost an average of 292 min (210 min-380 min), with an average of 436 mL (100 mL-1, 600 mL) blood loss. One patient (9.1%) died within 90 days after surgery, and another 4 cases (36.4%) suffered postoperative complications such as arrhythmia, cerebral infarction or hypoxemia. Six cases of squamous cell carcinoma, 4 cases of adenocarcinoma and 1 case of sarcomatoid carcinoma were identified by pathology. Seven cases were staged as pT4N0M0 while 4 cases were staged as pT4N1M0. Nine patients underwent adjuvant chemotherapy, and two patients underwent radiotherapy during follow-up. The overall follow-up time was 2 to 53 months, the 3-year disease-free survival rate was 30.7%, the median disease-free survival time was 31 months, the 3-year overall survival rate was 49.1% and the median overall survival time was 33 months.

**Conclusion:**

For selected patients of non-small cell lung cancer complicated with left atrial tumor thrombus, choose a reasonable surgical approach to resect both the tumor and the thrombus, strengthen the perioperative management and apply neoadjuvant/adjuvant radiotherapy and/or chemotherapy, might obtain satisfying prognosis.

现有观点认为对特定的局部晚期非小细胞肺癌（non-small cell lung cancer, NSCLC）病例，积极手术治疗可能改善患者预后^[1]^。NSCLC合并肺静脉、左房瘤栓的病例相对少见，文献中多以病例报告形式呈现^[2-4]^。在国际肺癌研究协会（International Association for the Study of Lung Cancer, IASLC）第八版TNM分期中，侵犯纵隔、心脏、大血管的NSCLC仍归为T4期^[5]^，总体分期在IIIa期以上，预期生存情况较差，并非传统意义上适宜接受手术治疗的群体。然而，这些病例又有其特殊性，如不进行手术干预，瘤栓脱落可能导致急性心衰或体循环栓塞等严重并发症，而同期行原发灶切除和瘤栓取出，有相当大比例可获得R0切除，配合术前新辅助或术后辅助放化疗，可能获得较好的疗效。我们总结了单中心的病例队列，汇报如下。

## 1 材料与方法

### 1.1 一般资料

自2006年8月-2017年7月，就诊于中日友好医院胸外科，确诊为NSCLC合并左房瘤栓，并行手术治疗病例共11例。其中男性7例，女性4例，就诊年龄41岁-68岁，平均57.9岁。有症状患者9例，其中咳嗽7例，咯血或痰中带血6例，胸闷憋气4例，心慌胸骨后疼痛1例，体重明显下降1例。本项回顾性研究获中日友好医院临床研究伦理委员会批准，患者术前均签署知情同意，相关资料可用于医学研究。

### 1.2 方法

#### 1.2.1 术前检查治疗

入院后行胸部增强电子计算机断层扫描（computed tomography, CT）检查明确为肺占位性病变伴肺静脉、左房受累。进一步完善实验室相关检查，完善头颅核磁共振成像（magnetic resonance imaging, MRI）/CT、颈部/锁骨上区域B超、腹部B超/CT、骨扫描或全身正电子发射计算机断层显像（positron emission tomography/computed tomography, PET-CT）除外远处转移，行肺功能及血气分析检查，如实测肺功能欠佳，建议行登楼实验或6分钟步行实验。早期常规行经胸壁超声心动图检查，评估瘤栓大小、位置、侵犯程度，近期于术前及术中常规行经食道超声心动检查。术前常规行气管镜检查，利于获得明确病理诊断。对影像学检查疑诊为颈部或锁骨上淋巴结转移的病例术前均行穿刺活检排除，对疑诊为纵隔淋巴结转移的病例，近期均在术前行经支气管镜超声引导下针吸活检（endobronchial ultrasound guided transbronchial needle aspiration, EBUS-TBNA）或纵隔镜检查除外N2转移。

对一般状况较好，东部肿瘤协作组（Eastern Cooperative Oncology Group, ECOG）评分0分-1分的患者，术前获得明确病理诊断，病理类型对放化疗相对敏感，且肿瘤负荷较高、估计R0切除困难的患者，建议行术前新辅助放化疗。其中鳞癌患者首选化疗方案为吉西他滨（1, 250 mg/m^2^, D1, D8）+铂类（顺铂75 mg/m^2^，卡铂按AUC值计算，D1），腺癌患者首选化疗方案为培美曲塞（500 mg/m^2^, D1）+铂类（顺铂75 mg/m^2^，卡铂按AUC值计算，D1），两周期新辅助化疗之间间隔3周，视患者耐受情况，新辅助化疗后1月安排手术。术前放疗剂量及与手术间隔时间由多学科讨论决定，一般总体剂量不超过60 Gy。

#### 1.2.2 手术操作及注意事项

术前需根据瘤栓大小、位置、侵犯程度决定是否行体外循环（cardiopulmonary bypass, CPB）或体外膜肺氧合（extracorporeal membrane oxygenation, ECMO）辅助，对术前评估瘤栓刚超过肺静脉基底部或预计心房壁受累小于1/6的病例，亦可选择侧卧位后外侧切口，或胸腔镜辅助小切口。

早期主要依据术前影像学检查除外合并可疑恶性胸腔积液的病例，近期选择先行患侧胸腔镜探查，以除外恶性胸腔积液及胸膜转移。无论选择正中开胸建立CPB，或者后外侧入路进胸，均应首先打开心包，探查心包内上、下腔静脉、左心耳、左房壁、上、下肺静脉有无肉眼浸润，判断所能达到的切除程度，如预计能达到R0/R1切除，可先行处理肺动脉、支气管，最后处理左房。对于侵犯左房程度较小（瘤栓刚超过肺静脉基底部或预计心房壁受累小于1/6）的病例，可选择应用胸腔镜Scanlan器械（Harken Clamp或DeBakey Clamp）夹闭左房后，行心房切开并取栓，再用4-0 prolene线对左房断面进行连续往返缝合，缝合完毕松开Scanlan钳，观察断面有无渗血或漏血，必要时用无创缝线八字加固缝合或用纱布压迫止血。对于侵犯左房范围较广（预计心房壁受累大于1/6）的病例，如有必要，可在CPB或ECMO辅助下，应用心脏外科技术作心房切面，必要时可用心包补片修补心房。注意避免因为盲目离断心房壁而造成瘤栓的不完整切除甚至脱落，切缘应远离左房顶，避免损伤发自右冠状动脉的窦房结动脉，导致术后窦房结功能障碍进而引起心律失常。

移除全肺/肺叶切除及心房壁/肺静脉瘤栓标本后，还应常规行纵隔淋巴结清扫，以获得更精确的病理分期，为术后治疗选择和预后判断提供支持。

#### 1.2.3 术后管理与随访

术后应密切监测血流动力学改变，定期行床旁超声心动或经食道超声心动检查了解心功能情况。术后宜尽早开始呼吸功能锻炼。肺癌合并瘤栓患者有明显的高凝倾向，在无活动性出血的条件下，应尽早开始低分子肝素抗凝。

根据患者术后恢复情况和病理分期选择术后辅助放化疗，化疗方案较前相同，一般行4个周期。术后定期进行门诊或电话随访，如有局部复发或转移征象，提请多学科讨论决定是否行放疗或其他辅助治疗。记录肿瘤复发和患者死亡日期，进行生存分析。

### 1.3 统计学分析

采用统计软件SPSS 19.0进行数据分析，计数资料采用率（%）表示。

## 2 结果

11例患者中，7例气管镜下可见病灶并获得明确病理诊断，2例行术前CT引导下穿刺获得病理诊断，2例术中送冰冻病理明确诊断。共有7例在术前接受PET-CT检查，4例疑诊纵隔淋巴结转移病例中3例行EBUS-TBNA，1例行纵隔镜活检可疑淋巴结，并除外N2转移。结合胸部增强CT及超声心动检查结果，考虑其中7例患者病变体积较大、侵犯左房程度较重，对其中身体状况尚佳的6位行术前新辅助放化疗。按照实体瘤疗效评价标准（Response Evaluation Criteria in Solid Tumors, RECIST），无完全缓解（complete response, CR）病例，2例获得部分缓解（partial response, PR），4例病灶略缩小或保持稳定（stable disease, SD），无疾病进展（progressive disease, PD）病例。

本组患者均顺利接受手术治疗，其中行正中开胸CPB下手术3例，ECMO辅助下侧卧位后外侧切口入路手术1例，常规侧卧位后外侧切口入路手术6例，侧卧位胸腔镜辅助小切口入路手术1例。手术达R0切除9例，R1切除2例，手术用时210 min-380 min，平均292 min，出血量100 mL-1, 600 mL，平均436 mL。全组有1例（9.1%）术后90天内死亡病例，患者术后合并心肺功能不全，插管上机后因合并严重肺部感染死亡，另有4例（36.4%）出现围术期并发症，包括心律失常2例，脑梗1例、低氧血症（可疑肺栓塞）1例，经治疗好转后均顺利出院。术后病理诊断鳞癌6例，腺癌4例，肉瘤样癌1例，术后病理分期pT4N0M0 7例，pT4N1M0 4例。术后对9例患者行辅助化疗。6例鳞癌患者中，2例术前新辅助化疗获得PR患者和2例未行新辅助化疗患者选择吉西他滨（1, 250 mg/m^2^, D1、D8）+铂类（顺铂75 mg/m^2^，卡铂按AUC值计算，D1）方案化疗4周期；1例SD患者更换为多西他赛（75 mg/m^2^, D1）+顺铂（75 mg/m^2^, D1）方案化疗4周期；1例患者因行左全肺切除，选择吉西他滨（1, 250 mg/m^2^, D1、D8）单药方案化疗。3例腺癌患者选择培美曲塞（500 mg/m^2^, D1）+铂类（顺铂75 mg/m^2^，卡铂按AUC值计算，D1）方案化疗4周期后，继续培美曲塞（500 mg/m^2^, D1）单药化疗方案长期维持。每两周期辅助化疗之间间隔3周。随访期内对1例局部复发和1例脑转移患者行放射治疗。

全组病例手术治疗及围术期情况见表 1。其中病例1术前、术后6月随访时CT如图 1、图 2所示。

**1:** 全组病例术式选择及围术期情况 The surgical treatment options and perioperative clinical characteristics

No.	Age	Sex	Extent of the left atrial invasion	Neoadjuvant treatment	Curative effect of neoadjuvant treatment	Procedure^*^	CPB/ ECMO	Degree of Resection	Duration of operation (min)	Blood loss (ml)	Pathology	pTNM staging	Perioperative complications	Adjuvant treatment	Prognosis
1	55	Male	Just beyond the base of pulmonary vein	-		VATS assisted LLL resection	-	R0	240	200	squamous carcinoma	pT4N1M0	cerebral infarction	chemotherapy	Alive, 53 months after surgery
2	63	Male	left atrial invasion less than 1/6	-		RUL resection	-	R0	300	350	Sarcomatoid carcinoma	pT4N0M0	-	-	Local recurrence at the 8^th^ month postoperatively. Dead, 14 months after surgery
3	61	Male	left atrial invasion more than 1/6	chemotherapy	SD	median sternotomy+ RML/RLL resection	CPB	R0	380	600	squamous carcinoma	pT4N0M0	-	chemotherapy	Dead, 33 months after surgery
4	47	Male	Just beyond the base of pulmonary vein	-		RML/RLL resection	-	R0	210	100	squamous carcinoma	pT4N0M0	-	chemotherapy	Alive, 35 months after surgery
5	41	Male	left atrial invasion more than 1/6	chemotherapy + radiotherapy	SD	median sternotomy+ left pneumonectomy	CPB	R0	360	400	squamous carcinoma	pT4N0M0	arrhythmia	chemotherapy	Dead, 22 months after surgery
6	58	Male	left atrial invasion more than 1/6	chemotherapy	SD	LUL resection	ECMO	R0	330	400	adenocarcinoma	pT4N0M0	hyoxemia	chemotherapy + radiotherapy	Found with Brain metastases at the 18^th^ month postoperatively. Dead, 25 months after surgery
7	62	Male	left atrial invasion more than 1/6	chemotherapy	PR	LUL resection	-	R0	270	200	squamous carcinoma	pT4N0M0	-	chemotherapy	Local recurrence at the 31^st^ month postoperatively. Dead, 38 months after surgery
8	66	Female	left atrial invasion less than 1/6	-		RML/RLL resection	-	R0	220	150	adenocarcinoma	pT4N1M0	-	chemotherapy	Alive, 15 months after surgery
9	68	Female	left atrial invasion more than 1/6	-		median sternotomy+LLL resection	CPB	R1	330	600	adenocarcinoma	pT4N1M0	heart failure, pulmonary infection	-	Dead, 2 months after surgery
10	59	Female	left atrial invasion more than 1/6	chemotherapy	PR	RUL/RML resection	-	R0	260	200	squamous carcinoma	pT4N0M0	-	chemotherapy	Alive, 36 months after surgery
11	57	Female	left atrial invasion more than 1/6	chemotherapy	SD	LLL resection	-	R1	310	1600	adenocarcinoma	pT4N1M0	arrhythmia	chemotherapy + radiotherapy	Local recurrence at the 16^th^ month postoperatively. Alive, 30 months after surgery
*RUL: right upper lobe; RML: right middle lobe; RLL: right lower lobe; LUL: left upper lobe; LLL: left lower lobe.															

**1:**
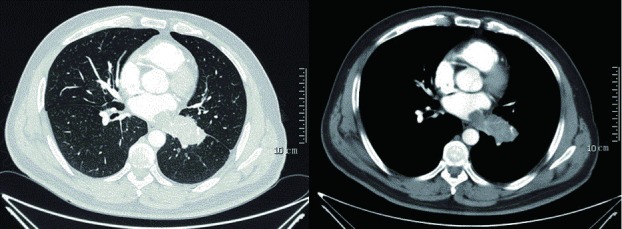
病例1术前胸部增强CT Preoperative chest CT image of case No.1

**2:**
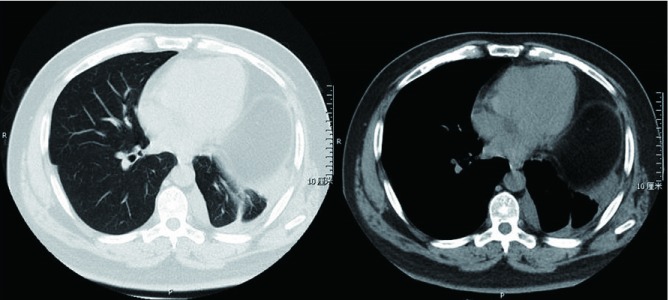
病例1术后6月随访胸部CT Postoperative chest CT image of case No.1 at 6-month follow up

全组患者术后定期门诊或电话随访，随访时间2个月-53个月。据随访情况绘制*Kaplan-Meier*生存曲线（图 3，图 4），得到3年无病生存率（disease-free survival, DFS）为30.7%，中位无病生存期31个月。3年总体生存率（overall survival, OS）为49.1%，中位总体生存期为33个月。

**3:**
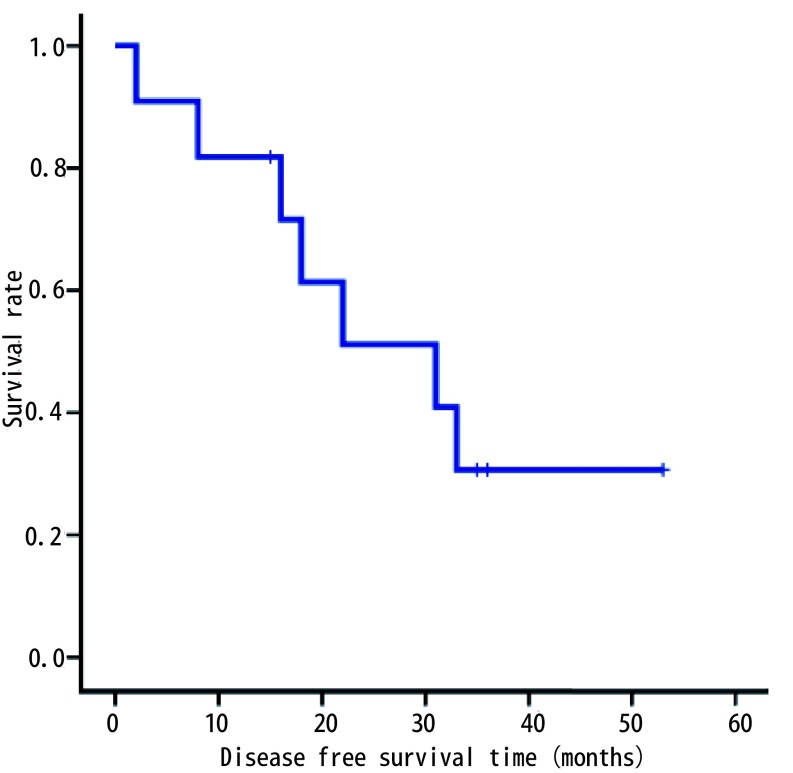
11例非小细胞肺癌合并左房瘤栓患者术后无病生存曲线 The disease-free survival curve of 11 cases of surgically treated non-small cell lung cancer with left atrial tumor thrombus

**4:**
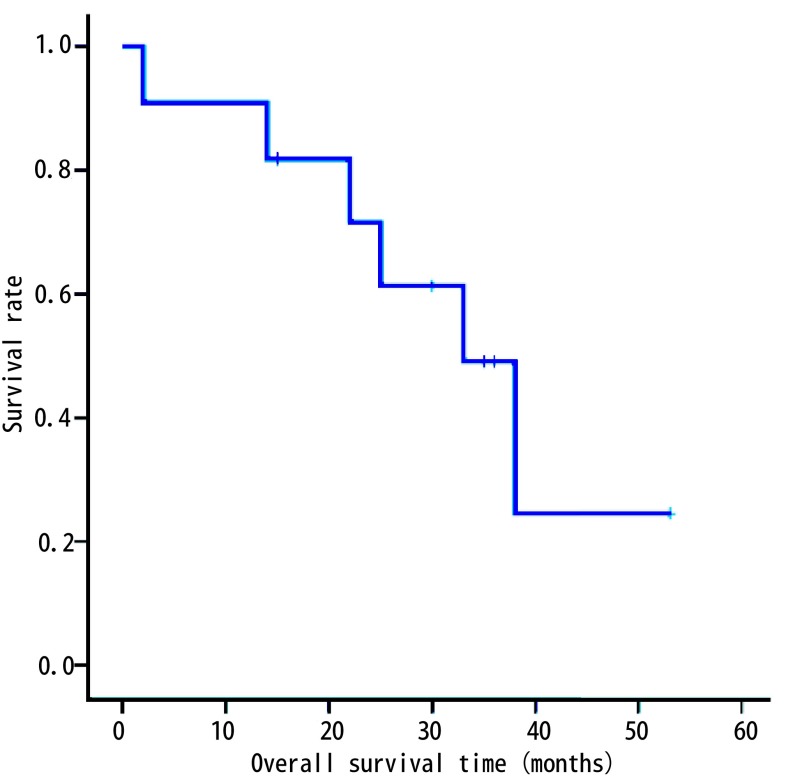
11例非小细胞肺癌合并左房瘤栓患者术后总体生存曲线 The overall survival curve of 11 cases of surgically treated non-small cell lung cancer with left atrial tumor thrombus

## 3 讨论

查阅文献，NSCLC合并左房受累行手术治疗以个案报道为主，少见大宗病例报道。各类NSCLC均可以瘤栓的形式累及左房，这其中以鳞癌^[6]^最常见，亦包括腺癌^[7]^、腺鳞癌^[4]^、甚至胚胎来源肿瘤^[8]^等。

疾病分期上，“侵犯纵隔、心脏、大血管”的NSCLC在IASLC第八版TNM分期中仍归为T4期5，总体分期在IIIa期以上。事实上，肿瘤直接浸润生长与通过瘤栓“抵达”肺静脉甚至左房在解剖和组织学上存在差异。该差异在一定程度上反映了肿瘤的生物学行为，是恶性程度高低的体现，可能对预后产生影响。与直接浸润生长相区别，瘤栓的延展范围相对局限，仅仅在有限空间（如血管腔）内增加体积，罕见浸润血管壁、心包等，恶性程度相对较低。在保证切缘阴性的条件下，通过手术完整移除可能获得较好的预后。现存分期尚不能体现其特殊性，未来在多中心、大样本、完善随访的基础上可能制定出更合理的T分期。

术前准备方面，应当强调检查的完备性。术前必须完善胸部增强CT和超声心动等检查，以判断病灶位置、左房大小及瘤栓侵及的范围、深度。对经胸壁超声观察不清晰的病例，可考虑在术前、术中行经食道超声心动检查，后者已被证实在各类复杂心脏疾病的诊断和术中监测、评价手术即刻效果方面有独特优势^[9]^，适用于合并有左房瘤栓的肺癌病例的术前诊断和术中监测。考虑到T分期较晚，本中心将纵隔淋巴结转移设定为肺癌合并左房瘤栓患者接受手术治疗的排除标准。对拟行手术治疗的患者，全部均经由多学科讨论，对一般状况较好（ECOG评分0分-1分），已知病理类型对放化疗敏感，且肿瘤负荷较高、估计R0切除困难的患者，建议行术前新辅助放化疗。

手术技术上，应合理评估是否需要CPB辅助。CPB下切除与肺癌合并肺静脉、左房瘤栓类似的局部晚期肺癌，能有效避免因肿瘤侵犯心脏、大血管所致的意外大出血，保证术中血流动力学稳定，使得常规方法不能切除的肿瘤得到完整切除，同时能减少因反复探查、剥离压迫肿瘤而导致的肿瘤血行播散的几率^[10, 11]^。此外，术中还可根据监测情况适时控制体温，降低代谢，对重要脏器有一定保护作用。需要注意的是，CPB的应用有严格的适应症，需以手术可达到根治为前提。而有观点认为CPB辅助肺癌根治及心脏、大血管部分切除术后并发症发生率高，围术期死亡率高，预后欠佳^[12]^。伴随体ECMO技术的成熟，ECMO可取代CBP适用于侵及肺静脉及左房的局部晚期肺癌^[13]^。而对估计心房壁受侵范围小于1/6或瘤栓刚超过肺静脉基底部和心房汇合处的病例，可以考虑不应用CPB或ECMO辅助^[14]^。本单中心病例队列中共有3例患者在CPB辅助下完成手术，2例出现术后并发症，其中1例因心功能不全、肺部感染于术后2个月死亡。提示CPB辅助可能是预后较差的危险因素，但样本量不足，且不能除外选择偏倚，如接受CPB辅助的患者病灶体积较大、分期较晚、新辅助化疗效果欠佳等。因此尚需大样本、前瞻性研究来比较CPB辅助下完成手术与其他术式，甚至保守治疗的疗效。

无论有无CPB或ECMO辅助，术中行心房切开和取栓都是手术的关键步骤，而应用心脏外科技术作心房切面的处理最为重要。需要避免因为盲目的结扎、离断心房壁或下肺静脉而造成瘤栓的不完整切除甚至脱落，进而导致体循环栓塞甚至急性心衰等致死性急症。需要特别指出的是，伴随腔镜技术的普及和发展，胸腔镜的应用对类似病例的诊疗有积极作用。一方面，对合并瘤栓的局部晚期肺癌患者，发生胸膜转移以致恶性心包、胸腔积液的可能性很大，先行胸腔镜探查有利于早期发现，从而避免探查性手术的发生；另一方面，胸腔镜对视野的放大作用有利于术中对肿瘤范围及侵犯程度的判定，避免了传统单纯靠术者“手摸”的情况，降低了因反复触碰肿瘤造成瘤栓脱落的风险。

围术期管理上，考虑到手术操作对心房结构及血流动力学的扰动，术后应密切监测血流动力学改变，定期行床旁超声心动或经食道超声心动检查了解心功能情况。术后宜尽早开始呼吸功能锻炼，加强气道管理。肿瘤患者存在高凝倾向，可检索到大量NSCLC患者术后发生急性肺栓塞^[15]^、体循环栓塞^[16]^的报道。即便应用预防性抗凝，对合并肿瘤瘤栓的病例，手术切除瘤栓后再发血栓栓塞的比例亦较高^[17]^。对这类患者，应着眼于预防大块肺栓塞、降低下肢大动脉栓塞发生几率和处理微小肺栓塞累积造成的肺动脉高压，同时注意抗凝治疗带来的出血风险。本组有术后出现新发脑梗和疑似肺栓塞诱发低氧血症的病例，证明类似肺癌合并瘤栓病例术后出现血栓栓塞的风险极高，积累经验后必要时可予更积极处理。

总体预后上，文献报道NSCLC合并肺静脉、左房瘤栓病例的预后尚佳。Spaggiari等^[18]^报道了15例非CPB下左房部分切除治疗NSCLC合并左房受侵病例，术后3年OS达到39%。Galvaing等^[19]^报道了19例非CPB下手术病例，其中16例接受术前新辅助放化疗，术后并发症发生率为52.6%，术后30天内死亡率10.5%，术后5年OS为43.7%。Ratto等^[20]^建议术前行PET-CT了解N分期，所报道19例患者中3例N2病例行术前辅助化疗，手术未达到R0切除或pN2病例接受术后放化疗，总体5年OS为14%，中位生存期25个月。本项研究未纳入N2病例，且新辅助/辅助放化疗选择由综合因素决定，得到术后90天内死亡率9.1%，围术期并发症发生率36.4%，3年DFS为30.7%，中位无病生存期31个月，3年OS为49.1%，中位总体生存期为33个月，提示手术治疗总体预后尚佳。

本项研究存在一些不足，NSCLC合并肺静脉、左房瘤栓病例相对少见，单中心样本量有限，故难免存在选择偏倚，且无法进一步行危险因素分层，获知影响预后的独立危险因素，仅能粗略统计生存率。尽管所得生存率相对乐观，提示对有选择的患者而言，包括手术、新辅助/辅助放化疗在内的综合治疗可能提供获益，该结论仍需要大样本、多中心的研究结果加以验证。
